# The excess costs of childhood food allergy on Canadian families: a cross-sectional study

**DOI:** 10.1186/s13223-021-00530-9

**Published:** 2021-03-10

**Authors:** Michael A. Golding, Elinor Simons, Elissa M. Abrams, Jennifer Gerdts, Jennifer L. P. Protudjer

**Affiliations:** 1 The Children’s Hospital Research Institute of Manitoba, 501G-715 McDermot Avenue, Winnipeg, MB R3M 3P4 Canada; 2 Department of Pediatrics and Child Health, The University of Manitoba, Winnipeg, MB Canada; 3 Division of Allergy & Immunology, Department of Pediatrics, Faculty of Medicine, The University of British Columbia, Vancouver, BC Canada; 4 Food Allergy Canada, Toronto, ON Canada; 5 George and Fay Yee Centre for Healthcare Innovation, Winnipeg, MB Canada; 6 Centre for Allergy Research, Karolinska Institutet, Stockholm, Sweden; 7 Food and Human Nutritional Sciences, The University of Manitoba, Winnipeg, MB Canada

**Keywords:** Costs, Food allergy, Pediatrics, Quality of life

## Abstract

**Background:**

The impact of childhood food allergy on household costs has not been examined in Canada. The current study sought to examine differences in direct, indirect, and intangible costs among Canadian families with and without a food-allergic child.

**Methods:**

Families with a child with a specialist-diagnosed food allergy (cases) were recruited from two tertiary pediatric allergy clinics in the Province of Manitoba, Canada, and matched, based on age and sex, to families without a food-allergic child (controls). Cost data for the two groups were collected via an adapted version of the Food Allergy Economic Questionnaire (FA-EcoQ). Consideration was given to income, defined as above vs. below the provincial annual median income.

**Results:**

Results from 35 matched case/control pairs revealed that while total household costs did not significantly differ between cases and controls, food-allergic families did incur higher direct costs ($12,455.69 vs. $10,078.93, p = 0.02), which were largely attributed to spending on food. In contrast, cases reported lower, but not statistically significant, total indirect costs compared to controls ($10,038.76 vs. $12,294.12, p = 0.06). Families also perceived their food-allergic child as having poorer quality of life relative to their healthy peers. Lastly, stratification of the analyses by annual income revealed several differences between the higher and lower income groups.

**Conclusions:**

Relative to families without a food-allergic child, food-allergic families incurred higher direct costs across a number of different areas.

**Supplementary Information:**

The online version contains supplementary material available at 10.1186/s13223-021-00530-9.

## Background

An estimated 6–8% of all children live with food allergy [1, 2], which has a considerable impact on the child and their family [2–4]. While the impact of childhood food allergy on quality of life is well described [3–6], researchers are just beginning to trace the economic consequences of this condition [7–9]. Relative to those without a food-allergic child, families with food-allergic children are burdened with the added responsibilities of avoiding allergens, attending regular appointments with healthcare professionals, and teaching their children how to manage their allergy independently. In a 2019 systematic review, Bilaver et al. [10] found that food allergy imposes a considerable economic burden on the healthcare system, and on families. The greatest proportion of costs shouldered by families with a food-allergic child stemmed from the wages, leisure time, and productivity lost as a result of managing a pediatric food allergy. However, out-of-pocket expenses were found to account for a sizeable proportion of the additional costs of food allergy as well. Not surprisingly, research suggests lower income families are disproportionately affected by the costs of pediatric food allergy [11–13], including the costs of epinephrine auto-injectors (EAIs) [11]. However, the economic impact of pediatric food allergy on lower income families is not fully understood.

The present literature on food allergy-related household costs comes primarily from Sweden [3, 14, 15], in which healthcare and school food programs are heavily subsidized, and from the United States [13, 16], where healthcare costs may be offset by private insurance and school food programs are not universal. The Canadian system reflects a mix of both government subsidies and private/workplace insurance [17]. At present, the literature has yet to investigate the impact of pediatric food allergy on household spending among Canadian families. As healthcare systems and the cost of living vary considerably between countries, an investigation of household spending among Canadian food-allergic families is warranted in order to quantify the financial burden they face.

In light of the gaps in the literature, the current study aims to investigate the impact of pediatric food allergy on household expenditures in Canada by comparing the direct, indirect, and intangible costs reported by families with and without a food-allergic child. Given the apparent disparities in the economic impact of pediatric food allergy, a particular focus will be placed on understanding how differences in income affect the magnitude and types of costs incurred by food-allergic families.

## Methods

Recruitment of participants took place from March 2019 to 10 March 2020 in Winnipeg, Manitoba, Canada (i.e. ending before the COVID-19 pandemic began). Specifically, cases, defined as families of children aged 0–17 years diagnosed by a pediatric allergist with at least one food allergy, were recruited from two tertiary pediatric asthma and allergy clinics. Controls, defined as families with no food allergy or other chronic conditions that would affect food choices, were recruited via the same allergy and asthma clinics, and from local schools, childcare centres, and social media (e.g. Facebook). All families needed to speak and read English comfortably as the recruitment materials, consent form and survey were in English.

### Measures

All participants completed an adapted version of the Food Allergy Economic Questionnaire (FA-EcoQ) [18]. The original English language FA-EcoQ is a validated self-report measure, designed to measure the direct, indirect, and intangible costs associated with managing a food allergy. To date, the measure has been translated into several languages and has been successfully adapted for use in a number of different countries [14, 18–20]. For the current study, several terms were modified to reflect the Canadian vernacular. For instance, references to day hospitals were replaced with the term “hospital” and the currency of reference was Canadian dollars (CAD) rather than British Pounds (₤). To limit participant burden, items that were irrelevant to the aims of the current study were omitted. As part of the questionnaire, participants completed questions regarding their sociodemographics, number of food allergies, types of symptoms, allergic comorbidities (i.e., eczema, atopic dermatitis, rhinitis, asthma), healthcare utilization, spending habits, and quality of life.

Based on the collected data, four broad outcome variables were created:

#### Total household costs

Calculated as the sum of the annual direct and indirect costs for the household.

##### Direct costs

Comprised of the costs paid by the household for medical and non-medical expenses. In the current study, these included costs related to medications, food, and travel to healthcare providers. Mileage costs were calculated by multiplying the number of kilometers travelled by $0.35. This value was selected based on the University of Manitoba’s mileage reimbursement rate and is consistent with the per-kilometer fuel and depreciation costs of a mid-size sedan as estimated by the Canadian Automobile Association [21]. In instances when cost data were measured discretely by a Likert scale, the mid-point of the category was used in the estimations.

##### Indirect costs

Include the costs associated with the loss of time and/or wages. Herein, indirect costs included those incurred by travelling to healthcare appointments and hospital admissions, waiting for and meeting with healthcare professionals, grocery shopping, preparing food, and seeking health-related information. They also included any wages that were lost in the pursuit of pediatric healthcare. Indirect costs were calculated by multiplying the number of hours lost by the after-tax hourly wage reported by the family member/s incurring the cost. When hours lost were measured on a discrete, Likert-style scale, the mid-point of the category was used in any estimations employing that variable. For family members who were unemployed, their time was valued at the provincial after-tax minimum wage. For individuals who indicated they were employed, but did not report their income, their wage was imputed with the average for that family member.

##### Intangible costs

Are costs that are not easily quantifiable. These included the responding parent’s well-being as well as their perceptions of their spouse’s and child’s well-being. Intangible costs also included a measure of income sufficiency, quantified as the difference between a family’s annual household income and the annual household income they deem to be sufficient.

### Statistical analysis

Prior to conducting the substantive analyses, case and control families were matched based on the age and sex of the reference child. Age matching was done in ± 2 year intervals. Descriptive statistics (n/N, %, mean ± standard deviation (SD)) were used to analyse the demographics of the sample. T-tests and chi-squared tests were used to assess the demographic similarity of cases and controls. For each of the outcome variables, cost estimates were derived using a series of linear multiple regression analyses, in which controls were the reference group. In order to control for the impact of allergic comorbidities, household income, and household composition on the cost estimates, annual after-tax household income, the number of individuals living in the household, and a binary variable that indexed whether the target child had been diagnosed with an allergic comorbidity (i.e., eczema, atopic dermatitis, rhinitis, asthma) were included as covariates in each of the regression models, with the exception of the models predicting income sufficiency. In these analyses, annual income was omitted from the aforementioned list of covariates. We then performed a sensitivity analysis, in which we considered the excess costs of food allergy, amongst cases, categorized at above or below the provincial median household income of $68,147 [22]. In each of the sensitivity analyses, the number of household members and the allergic comorbidity variable were included as covariates. All regression analyses employed the use of robust standard errors. Statistical significance was set a priori at α = 0.05.

## Results

### Participant characteristics

The final sample consisted of 35 (18/35 [51.43%] boys) matched case/control pairs (N = 70; Additional file 1). Children were, on average, 6.42 ± 0.76 years old among cases and 7.02 ± 0.80 among controls (p = 0.59). The average age of the parent completing the questionnaire was comparable between cases and controls (37.08 ± 1.07 vs, 37.67 ± 1.35, respectively). Family size was also comparable, with an average of 4 people per household. Overall, families reported a mean annual after-tax household income of $73,160.74 ± 3052.63, which was comparable between cases and controls ($74,676.20 vs. $71,554.29, respectively; p = 0.60). Amongst cases, peanut and tree nut allergies were most prevalent (82.86%).

### Excess costs amongst cases vs. controls

Total household costs did not significantly differ between cases and controls ($22,494.46 vs. $22,373.05, p = 0.94). However, allergic families did report greater overall direct costs compared to families without a food-allergic child ($12,455.69 vs. $10,078.93, p = 0.02; see Table 1). This difference was largely driven by spending on food as cases, on average, spent $2280.44 more on groceries and restaurant meals annually ($12,344.20 vs. $10,063.76, p = 0.03). Food- allergic families also spent more on travel to medical appointments ($64.86 vs. $19.61, p < 0.01) and on medications ($46.64 vs. $ − 4.43, p = 0.01)^1^ relative to controls.

**Table 1:** Multiple linear regression analyses predicting annual direct household costs

	Cases (n = 35)	Controls (n = 35)	Difference	95% CI of the Difference	*p*-value
Total annual direct costs	$12,455.69	$10,078.93	$2376.76	$328.90, $4424.62	0.02
Food costs	$12,344.20	$10,063.76	$2280.44	$230.10, $4330.78	0.03
Transportation costs	$64.86	$19.61	$45.25	$17.56, $72.94	< 0.01
Medication costs	$46.64	$ − 4.43	$51.07	$14.00, $88.14	0.01

Annual household income, number of household members, and allergic comorbidities status (i.e., presence vs. absence) are included as covariates in each of the models

*95% CI* 95th percent confidence interval

While the difference in overall indirect costs between case and control families fell above our α value ($10,038.76 vs. $12,294.12, p = 0.06), food-allergic families were found to incur significantly *lower* food shopping and preparation costs ($9373.09 vs. $11,931.53, p = 0.02; Table 2). In contrast, food-allergic families had higher, but not statistically significant differences for healthcare consultation costs ($238.47 vs. $131.41, p = 0.06), lost wages ($144.78 vs. $81.54, p = 0.51), and costs as a result of seeking healthcare-related information in print and online ($282.43 vs. $149.65, p = 0.08).

**Table 2:** Multiple linear regression analyses predicting annual indirect household costs

	Cases (n = 35)	Controls (n = 35)	Difference	95% CI of the Difference	*p*-value
Total annual indirect costs	$10,038.76	$12,294.12	$ − 2255.36	$ − 4553.10, $42.37	0.06
Food shopping and preparation costs	$9373.09	$11,931.53	$ − 2558.44	$ − 4763.57, $ − 353.32	0.02
Healthcare consultation costs	$238.47	$131.41	$107.06	$ − 6.53, $220.65	0.06
Research costs	$282.43	$149.65	$132.78	$ − 14.52, $280.08	0.08
Lost wages	$144.78	$81.54	$63.24	$ − 126.53, $253.01	0.51

Indirect costs refer to lost wages or time. For instance, indirect food shopping and preparation costs quantify the costs associated with the time lost as a result of food shopping and preparation. Annual household income, number of household members, and allergic comorbidities status (i.e., presence vs. absence) are included as covariates in each of the models

*95% CI* 95th percent confidence interval

Despite incurring greater direct costs in a number of areas, cases did not perceive the sufficiency of their income as significantly different from controls (see Additional file 2). Similarly, cases did not rate their quality of life, nor the quality of life of their spouse as significantly different from controls. Parents of food-allergic children did, however, perceive their child as having a lower quality of life relative to their healthy peers (ß =  − 0.96, 95% CI − 1.72, − 0.21; p = 0.01).

### Findings stratified by after-tax annual income

In order to untangle the impact of income on the current findings, a number of stratified analyses were conducted in which participants were divided into two groups: above (n = 39) vs. below (n = 31) the provincial median income. There were no significant differences in total costs between cases and controls across the higher and lower income strata (see Additional file 3). However, food-allergic families above the median income reported greater total direct costs in comparison to controls ($14,427.65 vs. $10,197.60, p = 0.01), a finding which was driven by higher annual food costs ($14,329.68 vs. $10,178.23, p = 0.02; see Table 3). In contrast, total direct costs and spending on food amongst cases below the median income did not differ significantly from controls. However, cases in the lower income stratum did report spending more on medical-related travel ($81.06 vs. $18.93, p = 0.004).

**Table 3:** Multiple linear regression analyses predicting annual direct household costs among participants falling above and below the provincial median annual income level

		Participants above the provincial median income level					Participants below the provincial median income level					
		Cases (n = 20)	Controls (n = 19)	Difference	95% CI of the difference	p-value	Cases (n = 15)		Controls (n = 16)	Difference	95% CI of the difference	p-value
Total annual direct costs		$14,427.65	$10,197.60	$4230.04	$963.44, $7496.65	0.01	$10,264.09		$9527.69	$736.40	$ − 1594.05, $3069.85	0.52
Food costs		$14,329.68	$10,178.23	$4151.44	$861.49, $7441.40	0.02	$10,142.41		$9510.14	$632.27	$ − 1692.52, $2957.05	0.58
Transportation costs		$46.43	$26.80	$19.63	$ − 18.28, $57.55	0.30	$81.06		$18.93	$62.14	$21.60, $102.67	0.004
Medication costs		$51.45	$ − 7.42	$58.96	$ − 2.63, $120.56	0.06	$40.62		$ − 1.37	$41.99	$ − 6.69, $90.68	0.09

Annual household income, number of household members, and allergic comorbidities status (i.e., presence vs. absence) are included as covariates in each of the models

*95% CI* 95th percent confidence interval

Across both income groups, differences in *total* indirect costs between cases and controls failed to reach statistical significance (Table 4). Significant differences were found, however, in a number of specific cost categories among those in the lower income stratum. In particular, cases below the median income reported spending more time consulting with healthcare professionals relative to controls ($227.42 vs. $59.58, p = 0.002). Cases in the lower income stratum also reported losing significantly more earnings as a result of their child’s healthcare visits compared to those in the control group ($109.97 vs. $15.65, p = 0.04). The same was not true, however, for cases above the median income level. Cases in the higher income stratum did, however, report spending less time shopping for and preparing food ($10,706.87 vs. $13,696.94, p = 0.06), although this effect was not statistically significant.

**Table 4:** Multiple linear regression analyses predicting annual indirect household costs among participants falling above and below the provincial median annual income level

		Participants above the provincial median income level					Participants below the provincial median income level					
		Cases (n = 20)	Controls (n = 19)	Difference	95% CI of the difference	p-value	Cases (n = 15)		Controls (n = 16)	Difference	95% CI of the difference	p-value
Total annual indirect costs		$11,473.86	$14,216.84	$ − 2742.98	$ − 5840.50, $354.54	0.08	$8435.37		$9720.20	$ − 1284.82	$ − 5643.47, $3073.82	0.55
Food costs		$10,706.87	$13,696.94	$ − 2990.07	$ − 6020.12, $39.97	0.06	$7880.69		$9567.01	$ − 1686.32	$ − 5888.96, $2516.33	0.42
Healthcare consultation costs		$235.13	$204.12	$31.01	$ − 164.09, $226.11	0.75	$227.42		$59.58	$167.84	$68.95, $266.73	0.002
Research costs		$364.74	$174.81	$189.94	$ − 104.39, $484.27	0.20	$217.29		$77.94	$139.34	$ − 62.63, $341.31	0.06
Lost wages		$167.12	$140.97	$26.15	$ − 337.71, $390.01	0.88	$109.97		$15.65	$94.31	$5.53, $183.09	0.04

Annual household income, number of household members, and allergic comorbidities status (i.e., presence vs. absence) are included as covariates in each of the models

*95% CI* 95th percent confidence interval

Similar to indirect costs, findings differed between strata in terms of the intangible costs incurred by families. (Table 5). In particular, parents of food-allergic children in the lower income stratum rated the well-being of their child as significantly lower than controls (ß =  − 1.36, 95% CI − 2.67, − 0.06; p = 0.04), but they did not rate their spouse’s well-being, nor their own, as significantly different from the non-food-allergic families. In contrast, cases in the higher income stratum did not rate their own well-being nor the well-being of their family members as significantly different from controls. Lastly, no significant differences emerged with regards to income sufficiency in either strata.

**Table 5:** Multiple linear regression analyses predicting intangible costs incurred by the household, responding parent, spouse, and child among participants falling above and below the provincial median annual income level

	Participants above the median income level			Participants below the median income level		
	ß	95% CI	p-value	ß	95% CI	p-value
Responding Parent Well-being						
Controls	Ref	Ref		Ref	Ref	
Cases	0.01	− 0.81, 0.82	0.98	− 0.06	− 0.86, 0.74	0.87
Spouse Well-being						
Controls	Ref	Ref		Ref	Ref	
Cases	− 0.58	− 1.17, 0.01	0.06	0.71	− 0.43, 1.85	0.21
Child Well-being						
Controls	Ref	Ref		Ref	Ref	
Cases	− 0.71	− 1.68, 0.26	0.15	− 1.36	− 2.67, − 0.06	0.04
Income sufficiency						
Controls	Ref	Ref		Ref	Ref	
Cases	$13,689.03	$ − 5475.06; $32,853.11	0.16	$ − 5031.31	$ − 12,192.16, $2129.53	0.16

Number of household members and allergic comorbidities status (i.e., presence vs. absence) are included as covariates in each of the models. Annual household income was also included as a covariate in each of the models predicting well-being. Income sufficiency quantifies the difference between a family’s actual income and the income they deem sufficient to meet their needs

*95% CI* 95th percent confidence interval

### The impact of income on household costs among food-allergic families

A series of regression analyses were used to investigate the impact of family income on both the magnitude and types of costs incurred by families with a food-allergic child. In each analysis, costs were compared among cases above vs. below the provincial median income level. While few significant differences emerged between the two groups, cases falling above the median income level did report higher annual direct costs ($13,722.33 vs. $10,752.63, p = 0.02) due to greater spending on food ($13,625.03 vs. $10,620.03, p = 0.02). It is important to note, however, that food-allergic families in the higher income stratum reported spending a lower proportion of their income on food (15% vs. 22%). Not surprisingly, higher income food-allergic families also reported greater income sufficiency relative to lower income families (β = 23,746.28, 95% CI 12,099.50, 35,393.07, p < 0.001). Differences in the remaining analyses failed to reach statistical significance (Tables 6, 7, 8).

**Table 6:** Multiple linear regression analyses predicting annual direct household costs from income level among food-allergic families

	Income above median (n = 20)	Income below median (n = 15)	Difference	95% CI of the difference	*p*-value
Total annual direct costs	$13,722.33	$10,752.63	$2969.70	$397.23, $5542.18	0.02
Food costs	$13,625.03	$10,620.03	$3005.00	$396.69, $5613.31	0.02
Transportation costs	$51.56	$95.11	$ − 43.55	$ − 116.30, $29.19	0.23
Medication costs	$45.74	$37.48	$8.25	$ − 44.94, $166.65	0.25

Number of household members and allergic comorbidities status (i.e., presence vs. absence) are included as covariates in each of the models

*95% CI* 95th percent confidence interval

**Table 7:** Multiple linear regression analyses predicting annual indirect household costs from income level among food-allergic families

	Income above median (n = 20)	Income below median (n = 15)	Difference	95% CI of the difference	*p*-value
Total annual indirect costs	$12,663.55	$9150.23	$3513.32	$ − 1373.48, $8400.12	0.15
Food costs	$11,737.86	$8515.96	$3221.90	$ − 1492.64, $7936.45	0.17
Healthcare consultation costs	$314.02	$222.47	$91.55	$ − 74.14, $257.23	0.27
Research costs	$370.56	$271.93	$98.62	$ − 197.69, $394.94	0.50
Lost wages	$241.10	$139.86	$101.24	$ − 140.40, $342.88	0.40

Number of household members and allergic comorbidities status (i.e., presence vs. absence) are included as covariates in each of the models

*95% CI* 95th percent confidence interval

**Table 8:** Multiple linear regression analyses predicting intangible costs incurred by the household, responding parent, spouse, and child from income level among food-allergic families

	ß	95%CI	p-value
Responding Parent Well-being			
Lower income	Ref	Ref	
Higher income	0.20	− 0.64, 1.03	0.63
Spouse Well-being			
Lower income	Ref	Ref	
Higher income	− 0.46	− 1.32, 0.40	0.29
Child Well-being			
Lower income	Ref	Ref	
Higher income	0.66	− 0.52, 1.83	0.26
Income sufficiency			
Lower income	Ref	Ref	
Higher income	$23,746.28	$12,099.50, $35,393.07	< 0.001

Number of household members and allergic comorbidities status (i.e., presence vs. absence) are included as covariates in each of the models. Income sufficiency quantifies the difference between a family’s actual income and the income they deem sufficient to meet their needs

*95% CI* 95th percent confidence interval

## Discussion

In this cross-sectional study of families with a food-allergic child matched by age and sex to non-food-allergic families, total annual direct costs were approximately 20% higher amongst food-allergic families, a difference that was largely driven by food costs. While differences in total annual indirect costs between cases and controls fell just above our α value, annual indirect food costs were significantly *lower* among food-allergic families. Intangible costs were higher for food-allergic children, but not for other family members. With consideration to income above or below the provincial median, differences in annual direct costs largely persisted amongst families above the median income only. In contrast, annual indirect cost differences between cases and controls were largely limited to those falling below the median income level.

In the present study, all types of direct costs were significantly higher amongst cases vs. controls, with cases spending approximately $2300 CAD more, out-of-pocket, annually. These costs are approximately half of those identified in a recent review article, in which food-allergic families were found to spend $4507 CAD (converted from $3339 USD on 2 July 2020) per year, out-of-pocket, when cost estimates were averaged across 4 samples and three studies [14, 23, 24]. These costs were largely attributed to the cost of living, but were also influenced to a lesser degree by spending on health-related travel, medications, and health insurance premiums [10, 14, 16, 23]. These differences highlight the need to consider excess food allergy-related costs within different settings, owing to differences in healthcare, social systems, and costs of living. This is the first Canadian study to capture food allergy-related costs. Although Canada has universal healthcare, we nonetheless found significant cost differences between cases and controls. Likewise, food costs differed significantly, a difference which was not seen in a Swedish study [14]. A main contributor to food cost differences in Canada, but not Sweden may be the Swedish universal school lunch program, in which students are provided, by law, a free hot daily lunch that complies with their medical dietary restrictions [25]. In Manitoba, some schools in economically disadvantaged areas do offer meal programs, although these are typically subsidized locally and are not mandated to comply with dietary restrictions.

Interestingly, while cases in the present study reported higher direct food costs, they were found to have lower costs related to food shopping/preparation relative to families without a food-allergic child. Admittedly, this finding is perplexing at first glance as it runs counter to Swedish research that has found both non-significant differences and greater indirect food costs among food-allergic families [14, 15]. However, it is possible that the lower indirect food costs reported by food-allergic families in the current study may reflect the greater time pressures faced by North American families [26]. Given this scarcity of leisure time, Canadian food-allergic families may rely more heavily on a narrow selection of food items, including prepackaged allergen-friendly products, that are known to be safe. While such a strategy should confer indirect cost savings by limiting the need for purchase decisions and preparation time, it would likely result in higher direct costs and more monotonous diets. Consistent with this reasoning, food-allergic families in the current study did report higher direct food costs relative to families without a food-allergic child.

Unlike previous studies, in which parents of children with food allergy, but not the children themselves, had a lower quality of life [14], our results point toward a significantly poorer quality of life for the child only. Although intangible costs have no measurable dollar cost per se, these costs are nonetheless an important part of food allergy-related excess costs. Intangible costs speak to the need for community education to normalize and support the condition and provide credible and easily accessible information to support those living with the condition.

Annual direct costs differed by food allergy status for families above, but not below the provincial median, with the exception of travel costs. Interestingly, results revealed a large difference in direct food costs between cases and controls in the higher income stratum, but the same effect was not found among lower income families. Arguably, differences in food costs between the income strata reflect the fact that higher income families often have more disposable income [27]. As such, they may be more inclined to purchase allergen-friendly products as a way to save preparation time and to ensure the safety of their food-allergic child. In contrast, lower income families may not be able to justify the increased costs of specialized allergen-friendly products and may be forced to purchase products that require more preparation or those that have precautionary allergen labelling.

Interestingly, while significant food cost differences between cases and controls only emerged among higher income families, several findings particular to the lower income stratum were also found. Specifically, lower income food-allergic families reported greater travel expenses, healthcare consultation costs, and lost wages as result of their child’s healthcare visits, relative to families without a food-allergic child. The same differences, however, were not found among higher income families. Arguably, this discrepancy may reflect the fact that lower wage workers are not only less likely to have paid time off, but also have lower rates of vehicle ownership [28–30]. Moreover, we recently reported that a small proportion of mothers in high-income households reported career limitations, because of their child’s food allergy, which may provide insight into the lack of difference in lost wages between high-income families vs. controls [31].

While medication cost differences between cases and controls were not significant in either stratum, it is interesting to note that the amount that food-allergic families spent on medication was comparable regardless of income status ($51.45 vs $40.62, above and below the median, respectively). This observation is highly concerning as these amounts are less than half of the out-of-pocket expense for a single EAI in Manitoba. Whereas some families may have supplementary insurance to offset these out-of-pocket costs, it is also possible that families are not renewing their EAI prescriptions on an annual basis due to the high cost. Elsewhere, it has been reported that only about half of Canadians with food allergy have access to an EAI [32]. Whereas there are many hypotheses for low EAI carriage, our data do point toward a likelihood that costs may be a contributing factor. For families with incomes above the annual median, out-of-pocket medication costs total about 0.36% of their total direct costs. Families with incomes below the annual median spend approximately 11% more on out-of-pocket medications, for a total of 0.4%. Moreover, our observation highlights the need for federal and/or provincial funding to cover the costs of this potentially life-saving medication. In Canada, the rates of allergy-related emergency department visits, including anaphylaxis, have increased over the past decade [32]. In contrast, corresponding rates decreased in Sweden in the two years following the elimination of co-payments for EAIs for pediatric patients [33]. Whereas it was beyond the scope of the Swedish study to perform a cost-effectiveness analysis of the elimination of co-payments, we are confident that the costs absorbed by the healthcare system for EAIs are dramatically less than those associated with allergy-related hospitalization.

With consideration to costs amongst cases above vs. below the annual median, no significant differences were found in terms of indirect costs; however, food-allergic families above the median income were found to have significantly higher direct costs, a finding that was largely attributed to greater spending on food. It is important to note, however, that food-allergic families below the median income level devoted a greater proportion of their income to food. The financial burden faced by low income, food-allergic families, and the associated coping mechanisms, have been previously described qualitatively. In Canada, economically disadvantaged food-allergic families, report having to procure their food from discount supermarkets and food banks where they face concerns with cross-contamination [11]. Moreover, low-income food allergic families also report difficulties maintaining a nutritious diet over and above their allergy-related dietary requirements [11].

This is the first study to systematically examine the excess costs associated with food allergy, with consideration to income status, and to subsequently compare food allergy-related costs between economically disadvantaged vs. advantaged families. All food-allergic children in this study had been diagnosed with food allergy by a pediatric allergist prior to completing the FA-EcoQ questionnaire. As such, their families have had time to incorporate the necessary changes and reflect on the excess costs of food allergy. Finally, we highlight that we made use of a validated questionnaire [18], with which our group has previous experience [14, 15], but which was situationally adapted for use in this study population.

We acknowledge the limitations of this study, including that the mean incomes reported by cases and controls were slightly (but not significantly) higher than the median provincial income. Similarly, we lacked power to consider the costs amongst low-income Manitobans, defined by national cut-offs [34]. Finally, we applied some estimates when analyzing certain costs. For example, we estimated mileage costs when calculating travel to healthcare providers, who are based in Winnipeg, the largest urban centre in our province, and which is approximately 100 km from the border with the United States. As such, healthcare delivery in our province covers a vast catchment area, of approximately 650,000 km^2^ [35]. Consequently, rural-dwelling families may incur additional costs when accessing healthcare, including food and lodging, which were not available in the current dataset. Given the lack of food and lodging data, it is possible that the current study may have underestimated the actual travel costs incurred by rural families. Rural food-allergic families may have been particularly affected by this underestimation given the relatively low number of allergists in Canada and the consequent need for travel [32, 36].

The current study holds the potential to inform policy and programs aimed at supporting families burdened with the added costs of caring for a food-allergic child. Our results on reported income insufficiency amongst cases below the median income point toward a need for financial support for these families. There is a need to open discussions for health coverage for potentially life-saving medications, including EAIs, as well as tax credits for allergy-friendly foods for those with allergist-diagnosed food allergy. Notably, our cost estimates were based on data collected in the year prior to the COVID-19 pandemic. Going forward, it will be important to examine the impact of the pandemic on families’ abilities to absorb food allergy-related costs.

In conclusion, results suggest that compared to families without a food-allergic child, food-allergic families assume greater costs in a number of different areas. As spending on food accounts for much of the difference in direct costs between cases and controls, future researchers should strive to determine what products and practices are responsible for the differences in food spending between food-allergic and non-food-allergic families. Moreover, policy makers should consider how to alleviate this burden for those most adversely affected.

## Supplementary Information

**Additional file 1.** Atopic conditions. Frequencies of atopic conditions among cases and controls (N=70 case-control pairs). Table outlining the frequencies of various food allergies and atopic comorbidities.

**Additional file 2.** Intangible costs. Multiple linear regression analyses predicting intangible costs incurred by the household, responding parent, spouse, and child. Table displaying the results from a series of multiple linear regression analyses predicting intangible costs among cases and controls.

**Additional file 3. **Total costs stratified by income. Multiple linear regression analyses predicting total annual household costs among participants falling above and below the provincial median annual income level. Table displaying the results from a series of income-stratified linear regression analyses predicting total household costs among cases and controls.

## Data Availability

Datasets generated and analyzed during the current study are not publically available out of respect for participant privacy, but are available from the corresponding author upon reasonable request.

## Abbreviations

CAD: Canadian dollars
EAI: Epinephrine auto-injector
USD: United States (American) Dollars

## Footnotes

1 Medications costs for the control group are negative at the mean level of the covariates for each of the analyses predicting prescription costs. Consequently, the estimated marginal means of the medication costs for the control group should be interpreted with caution as they are likely not meaningful.
